# Country transition index based on hierarchical clustering to predict next COVID-19 waves

**DOI:** 10.1038/s41598-021-94661-z

**Published:** 2021-07-27

**Authors:** Ricardo A. Rios, Tatiane Nogueira, Danilo B. Coimbra, Tiago J. S. Lopes, Ajith Abraham, Rodrigo F. de Mello

**Affiliations:** 1 Institute of Computing, Federal University of Bahia, Salvador, Brazil; 2 Department of Reproductive Biology, National Center for Child Health and Development Research Institute, Tokyo, Japan; 3 Machine Intelligence Research Labs, Auburn, USA; 4 Institute of Mathematical and Computer Sciences, University of São Paulo, São Carlos, Brazil; 5 Present Address: Itaú Unibanco, Av. Eng. Armando de Arruda Pereira, São Paulo, Brazil

**Keywords:** Computer science, Computational science, Scientific data

## Abstract

COVID-19 has widely spread around the world, impacting the health systems of several countries in addition to the collateral damage that societies will face in the next years. Although the comparison between countries is essential for controlling this disease, the main challenge is the fact of countries are not simultaneously affected by the virus. Therefore, from the COVID-19 dataset by the Johns Hopkins University Center for Systems Science and Engineering, we present a temporal analysis on the number of new cases and deaths among countries using artificial intelligence. Our approach incrementally models the cases using a hierarchical clustering that emphasizes country transitions between infection groups over time. Then, one can compare the current situation of a country against others that have already faced previous waves. By using our approach, we designed a transition index to estimate the most probable countries’ movements between infectious groups to predict next wave trends. We draw two important conclusions: (1) we show the historical infection path taken by specific countries and emphasize changing points that occur when countries move between clusters with small, medium, or large number of cases; (2) we estimate new waves for specific countries using the transition index.

## Introduction

In December 2019, a new disease referred to as COVID-19 (Coronavirus disease 2019) was reported in Wuhan, China, and since then it has been spreading globally, leading the World Health Organization (WHO) to declare it a pandemic outbreak on March 11th, 2020^1^ (https://www.who.int/emergencies/diseases/novel-coronavirus-2019/interactive-timeline). COVID-19 is an infectious disease caused by severe acute respiratory syndrome coronavirus 2 (SARS-CoV-2) whose symptomatic cases include fever, cough, fatigue, and shortness of breath^2,3^. Although asymptomatic cases do not require special medical care, the scientific community has been trying to understand their influence in the pandemic, that is whether they act as important and silent vectors of person-to-person transmission^4–7^ or their immune systems are able to rapidly neutralize the virus^8,9^.

The great impact of COVID-19 has motivated the Johns Hopkins University Center for Systems Science and Engineering (JHU CSSE) to put together an online repository listing the number of new cases and deaths^10^, referred in this manuscript to as COVID-19-CSSE (COVID-19 Data Repository by the Center for Systems Science and Engineering (CSSE) at Johns Hopkins University available at https://github.com/CSSEGISandData/COVID-19). Such a repository includes reports from different institutions such as the World Health Organization (WHO) and local health agencies from different countries like China, Taiwan, United States, Australia, Singapore, Italy, France, and Israel. The COVID-19-CSSE repository has motivated us to model how this virus spreads in order to represent its impact in the most affected countries along time.

Our approach employs a hierarchical clustering algorithm, an unsupervised learning branch from the Artificial Intelligence, along with the average-link strategy^11^ to determine the pertinence of countries to groups along weeks to analyze how the disease spreads and affects different societies. Clustering partitions were evaluated using the mean silhouette^12,13^ to ensure modeling representability. We propose a transition index to estimate the most probable countries’ movements between infectious groups along weeks, helping to identify next waves. In summary, our study demonstrates that the usage of known machine learning methods is a feasible approach to model the spread of COVID-19. We anticipate that our results, together with other studies from the same scientific context^14–19^, will aid policymakers to implement guidelines and procedures derived from evidence that takes into account the global dynamics of infectious diseases.

## Results

To illustrate our approach, we consider the first death registered in Brazil (March 17th, 2020) until October 07th, 2020, so all countries having historical data before such date are taken into account (more details about the data organization is presented in Sections *Data Processing* and *Our Approach*). From this perspective, we reduced the COVID-19-CSSE dataset from 187 to 54 countries to study the infection trends in Brazil. We performed an empirical clustering analysis using the mean silhouette ($S_\mu$) to improve the cut off point for all dendrograms.

Finally, we cut dendrograms to form partitions with 3 groups (low, medium, and high disease incidences), simplifying our analysis while respecting the literature recommendation^12^: reasonable structure ($0.51 \le S_\mu \le 0.7$), and strong structure ($0.71 \le S_\mu \le 1$). In our context, the mean silhouette is not considered to find the optimal number of clusters. In turn, it is used to justify that our conclusions are not drawn from weak or non-substantial structures. Moreover, possible outliers are not removed from our analyses, once they are useful to, for example, track the current and next COVID-19 epicenters.

### Confirmed cases

**Figure 1:**
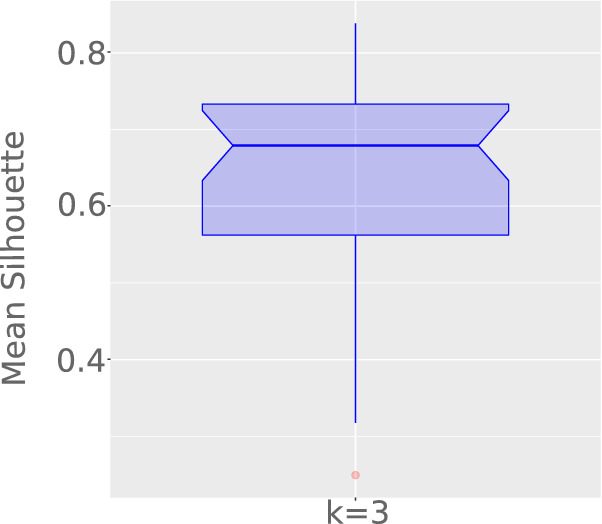
Mean Silhouette variation ($S_\mu$), considering three groups of countries clustered by the absolute number of confirmed cases per million inhabitants.

The first analysis involved the number of daily confirmed cases per million inhabitants. Figure 1 illustrates the mean silhouette along time, confirming an average around 0.68 and containing both central quantiles above 0.56. Results suggest partitions are representative for our problem.

**Figure 2:**
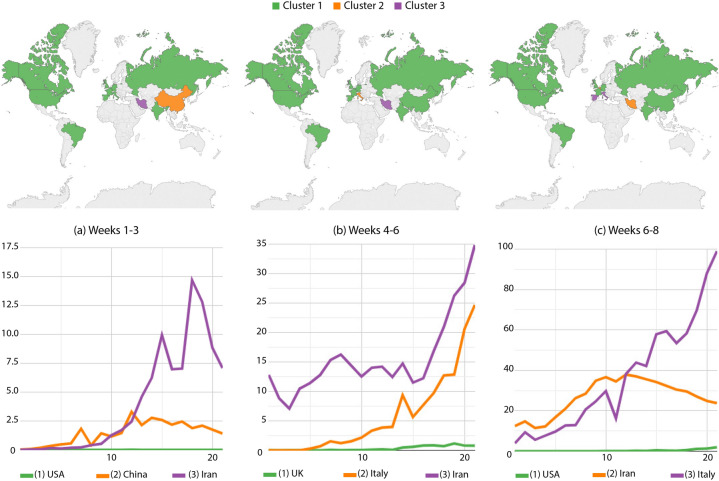
Confirmed cases per million inhabitants: country partitions along weeks 1–8. Top images identify country groups and bottom curves correspond to the infection incidence levels: green, orange and purple correspondingly map the low, medium and high-incidence groups. Curve legends indicate the group descriptor. Top-most maps were generated by using Tableau Desktop-Professional Edition (https://www.tableau.com/, version 20181.20.0213.2110-64 bit), and bottom-most charts were generated by using Google Charts (https://developers.google.com/chart, version 49).

**Figure 3:**
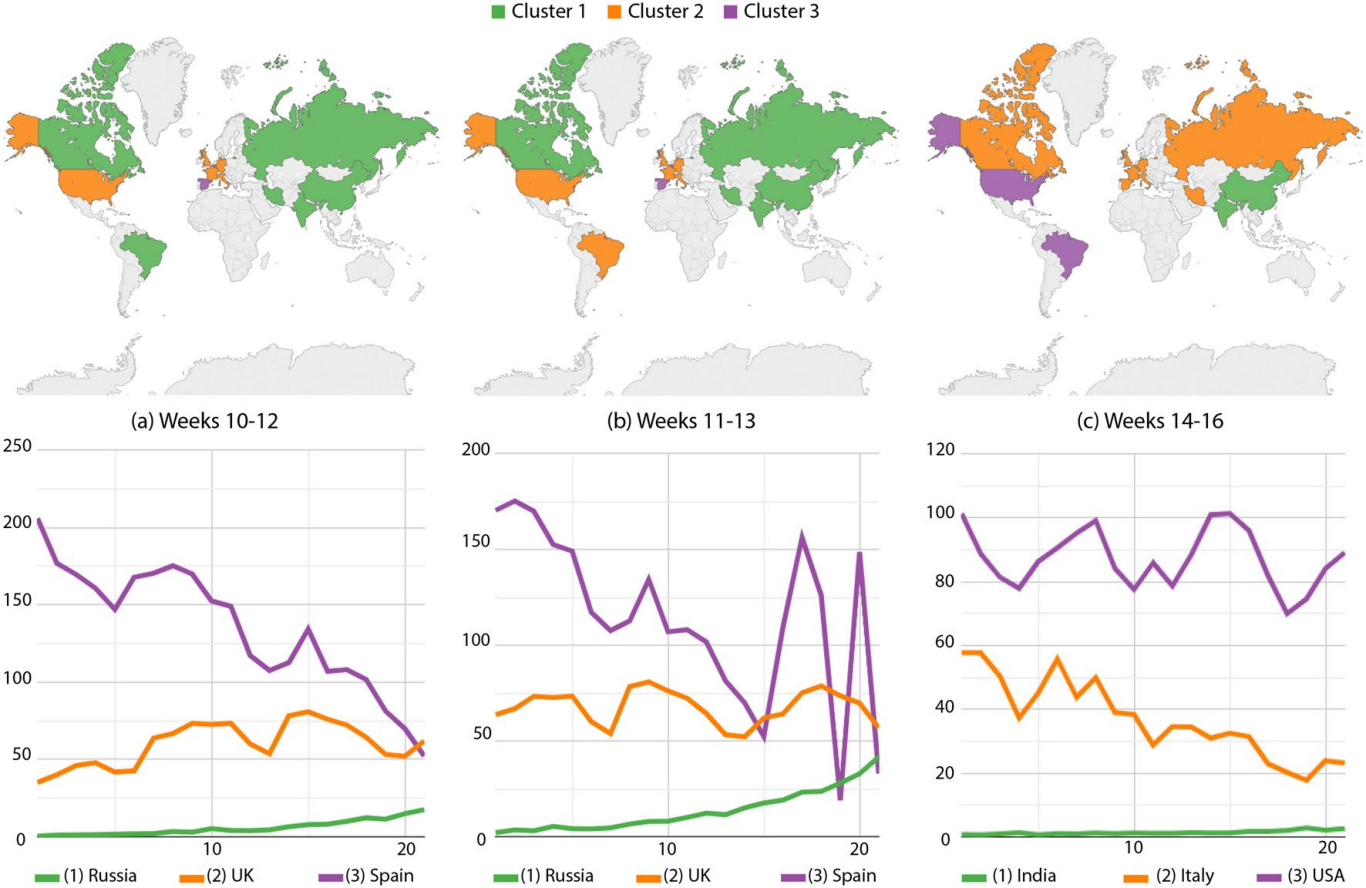
Confirmed cases per million inhabitants: country partitions along weeks 10–16. Top images identify country groups and bottom curves correspond to the infection incidence levels: green, orange and purple correspondingly map the low, medium and high-incidence groups. Curve legends indicate the group descriptor. Top-most maps were generated by using Tableau Desktop-Professional Edition (https://www.tableau.com/, version 20181.20.0213.2110-64 bit), and bottom-most charts were generated by using Google Charts (https://developers.google.com/chart, version 49).

**Figure 4:**
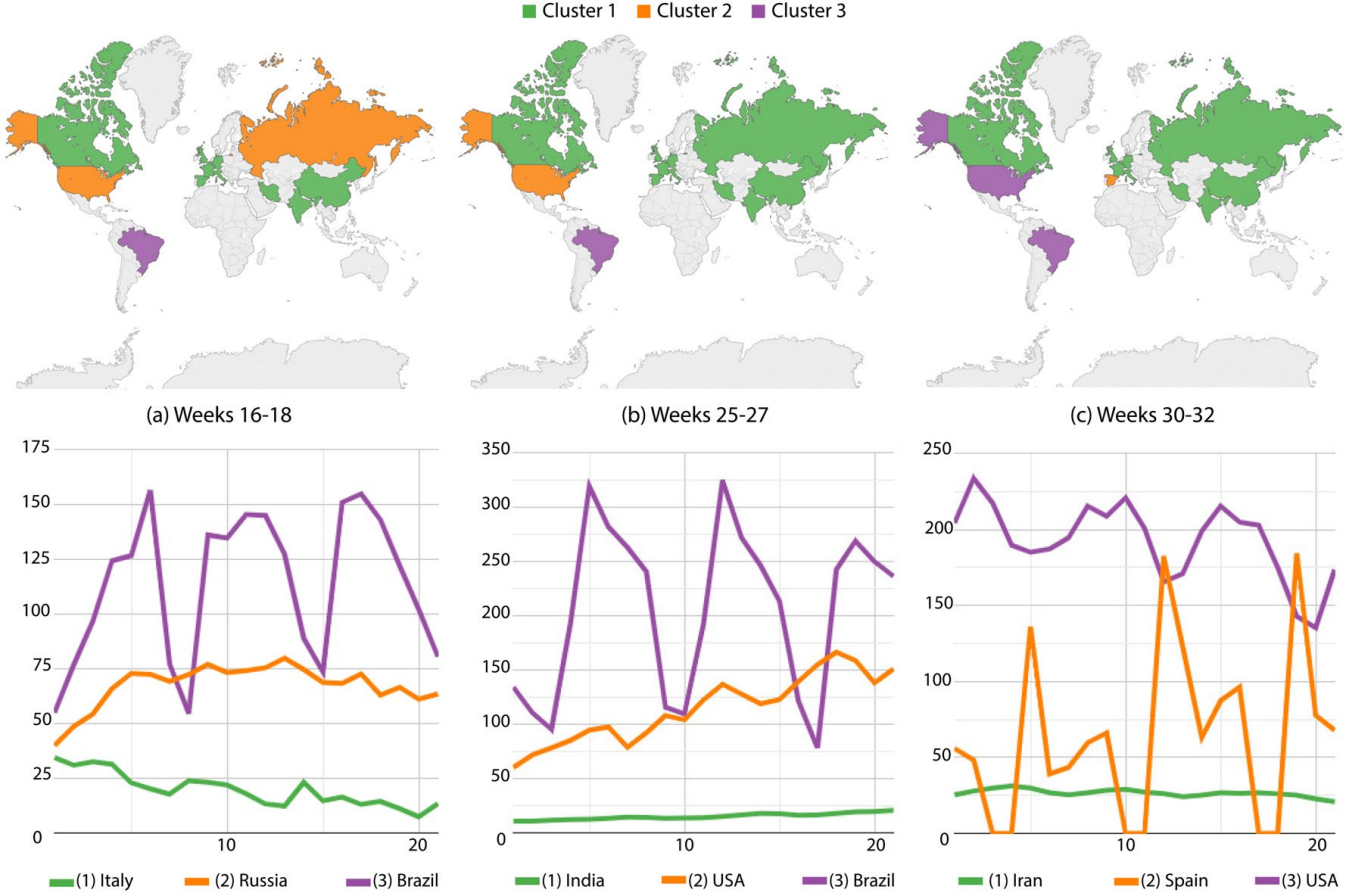
Confirmed cases per million inhabitants: country partitions along weeks 16–32. Top images identify country groups and bottom curves correspond to the infection incidence levels: green, orange and purple correspondingly map the low, medium and high-incidence groups. Curve legends indicate the group descriptor. Top-most maps were generated by using Tableau Desktop-Professional Edition (https://www.tableau.com/, version 20181.20.0213.2110-64 bit), and bottom-most charts were generated by using Google Charts (https://developers.google.com/chart, version 49).

By considering 3-week windows, sliding a week per iteration, our approach analyzed 35 intervals. Figures 2, 3 and 4 display World choropleth maps with the most relevant partitions along time, helping us to identify drift scenarios. From Fig. 2a,b, China leaves out the intermediary-incidence group, Italy joined that cluster, and Iran moved to the highest-incidence one. Figure 2c confirms Italy and Spain in the highest-incidence group, while Belgium and Iran move to the intermediary level and no change was performed by other countries. At the bottom curves, medoid countries or group descriptors are shown, i.e., countries better representing groups.

In Fig. 3a, Italy moved to the intermediary group while Belgium joined with Spain in the highest incidence. Furthermore, there is a reduction trend in this high-incidence group over time. Figure 3b confirmed Brazil, the USA, and the greater part of Western Europe in the intermediary group, while Belgium and Spain kept the higher incidences however under greater variations (see curves at the bottom of such figure). Brazil and the USA moved to the highest incidence group in Fig. 3c, while Canada, Iran and Russia joined most of the Western Europe (including Spain and Belgium) in the intermediary group. India and China maintained the smallest number of cases.

From weeks 16 to 18, Brazil was isolated in the worst group (Fig. 4a), while Russia and the USA were in the intermediary level. Canada, Western Europe, Iran, India and China somehow managed to reduced the contagion and kept the smallest numbers. Next, Russia moved to the best group while the others remain unchanged along weeks 25–27. The USA joined Brazil back in the worst-incidence group, while cases increase in Spain making it move back to the intermediary group.

From those drifts, we also suggest the interpretation of case trends using curves below World maps. There are some clear increases, decreases and stabilities to mention: Iran going up along Fig. 2a,b while decreasing in Fig. 2c; Spain and Belgium significantly decreasing along weeks 10–12 as seen in Fig. 3a; finally, many bumps in Brazil from weeks 16 to 18 and 25 to 27, and in Spain from weeks 30 to 32, a clear result of measurement discontinuities once cases were only accurately reported during business days^20^.

### Death cases

**Figure 5:**
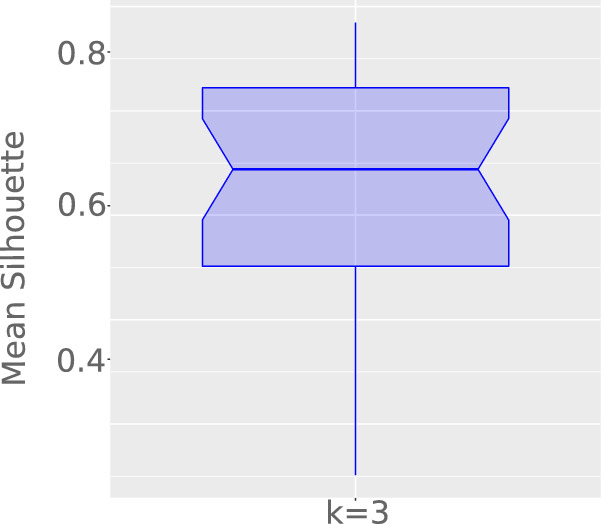
Mean Silhouette ($S_\mu$) variation by considering three groups of countries clustered by the absolute number of death per million inhabitants.

This second analysis involved the number of daily deaths per million inhabitants. We also analyzed the mean silhouette for partitions with three groups (Fig. 5), from which we obtained an average around 0.68 with both central quantiles above 0.56. Results suggest partitions are representative for our problem.

**Figure 6:**
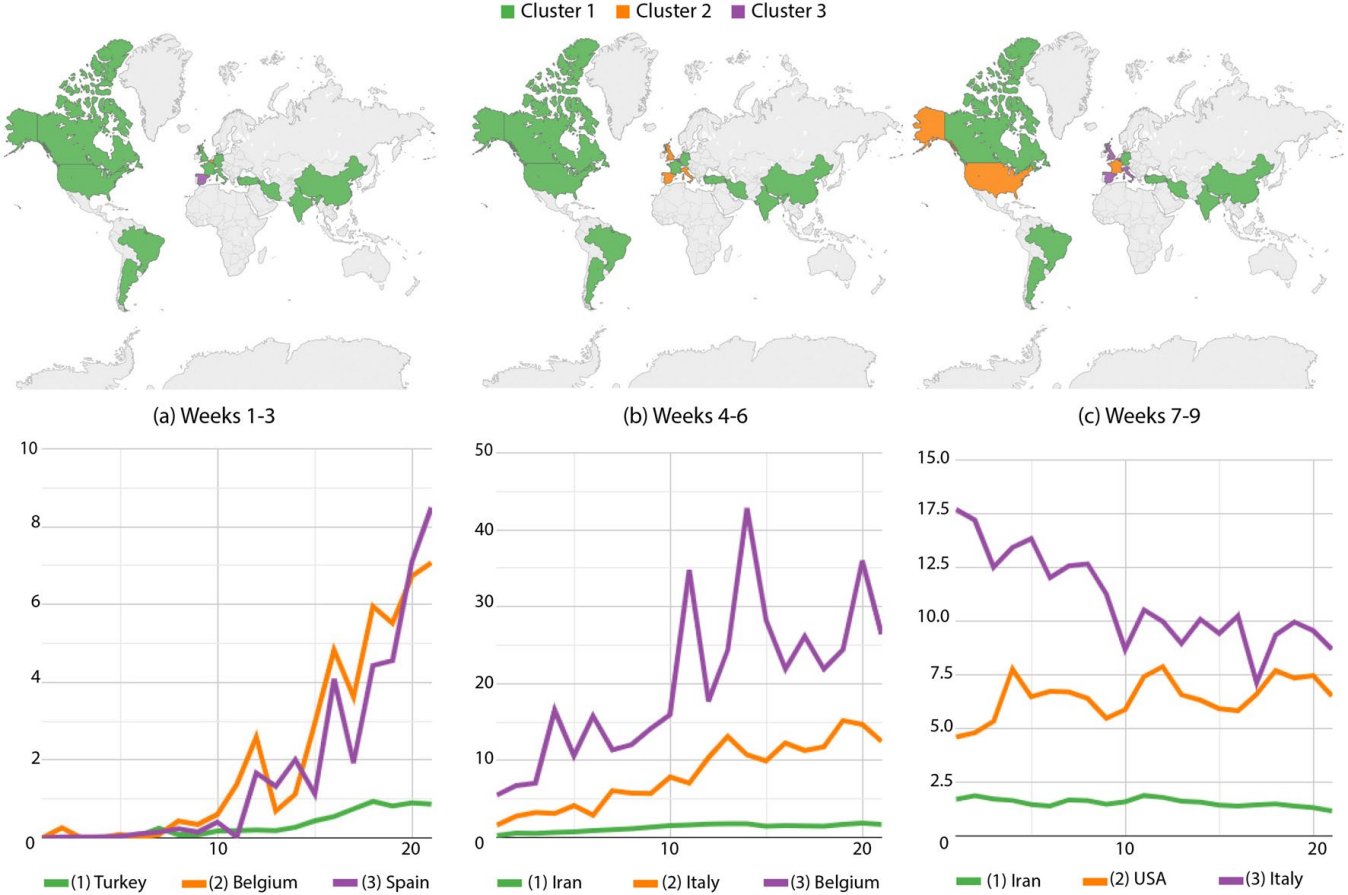
Death cases per million inhabitants: country partitions along weeks 1–9. Top images identify country groups and bottom curves correspond to the infection incidence levels: green, orange and purple correspondingly map the low, medium and high-incidence groups. Curve legends indicate the group descriptor. Top-most maps were generated by using Tableau Desktop-Professional Edition (https://www.tableau.com/, version 20181.20.0213.2110-64 bit), and bottom-most charts were generated by using Google Charts (https://developers.google.com/chart, version 49).

From weeks 1 to 3, Spain and Belgium got isolated into two groups with the highest incidences (Fig. 6a), while the remaining countries were still with small numbers. Trends of Spain and Belgium are very steep what most certainly confirms the motivation for the distancing policy adopted by their governments.

Belgium keeps increasing its death numbers from weeks 4 to 6 (Fig. 6b) at a greater pace than Spain, Italy and the UK, being all three located at the intermediary group. Then UK, Spain and Italy moved to the highest-incidence group with Belgium (Fig. 6c). France and the USA took over the intermediary group, depicting a relevant increase in death counts.

**Figure 7:**
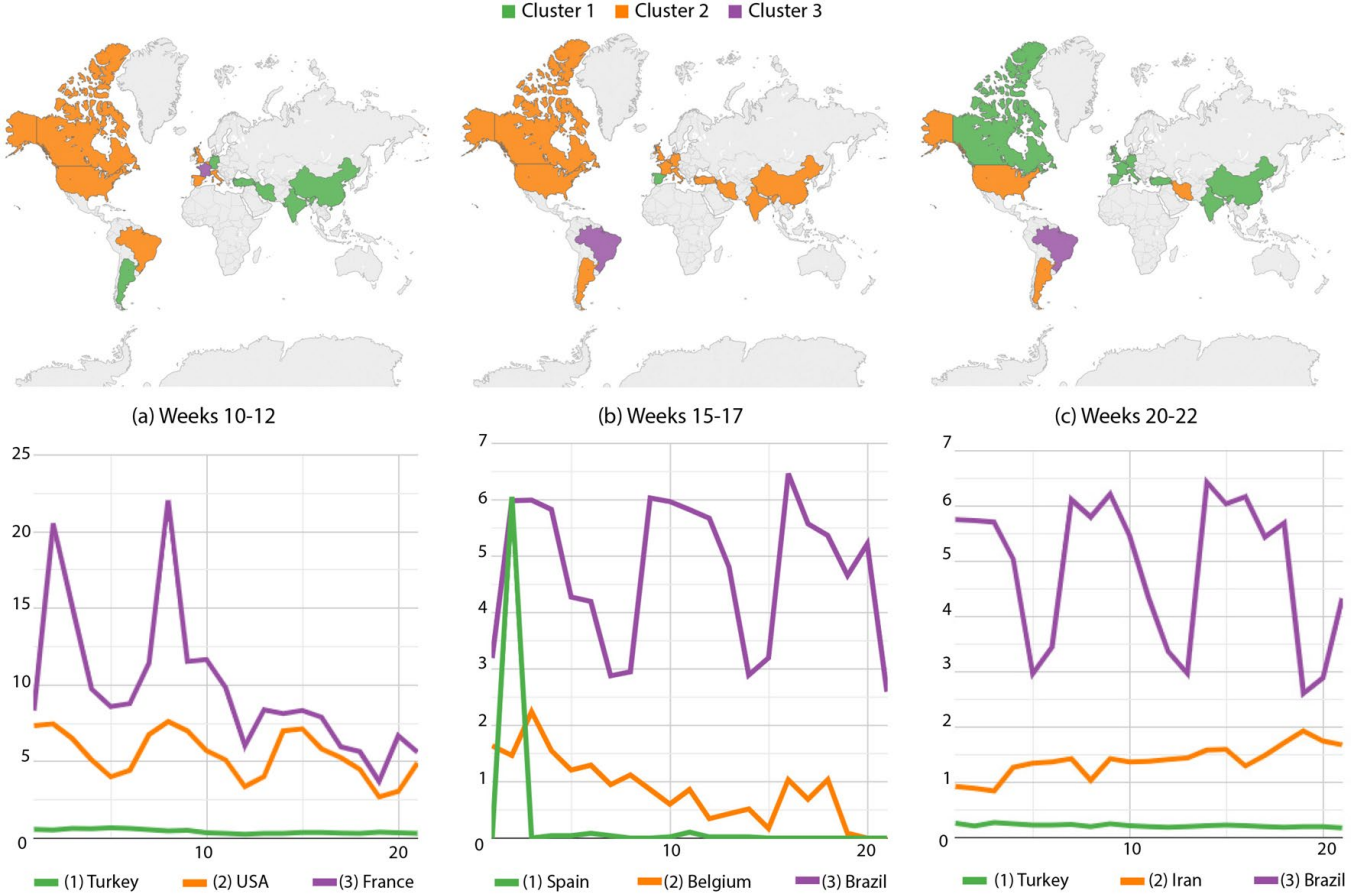
Death cases per million inhabitants: country partitions along weeks 10–22. Top images identify country groups and bottom curves correspond to the infection incidence levels: green, orange and purple correspondingly map the low, medium and high-incidence groups. Curve legends indicate the group descriptor. Top-most maps were generated by using Tableau Desktop-Professional Edition (https://www.tableau.com/, version 20181.20.0213.2110-64 bit), and bottom-most charts were generated by using Google Charts (https://developers.google.com/chart, version 49).

Along weeks 10–12, Argentina started participating in the less affected group. Brazil, Spain, Italy, Belgium, the UK and the USA composed the intermediary group. France took over the group with the highest incidence, besides its trend approaches the intermediary group (Fig. 7a). Next, from weeks 15 to 17 (Fig. 7b), Spain decreased its numbers, participating in the lowest-incidence group; meanwhile, Belgium, Argentina, Canada, the USA, most of the Western Europe, Turkey, Iran, India and China were at the intermediary level. Brazil started its upward trend by taking over the highest-incidence group.

From weeks 20 to 22, Canada, the Western Europe, Turkey, India and China had the smallest indices (Fig. 7c). Argentina, the USA and Iran were clustered together in the intermediary level, while the highest incidences were still on Brazil. We again noticed the bumpy Brazilian curve associated to less accurate reports at the weekends.

**Figure 8:**
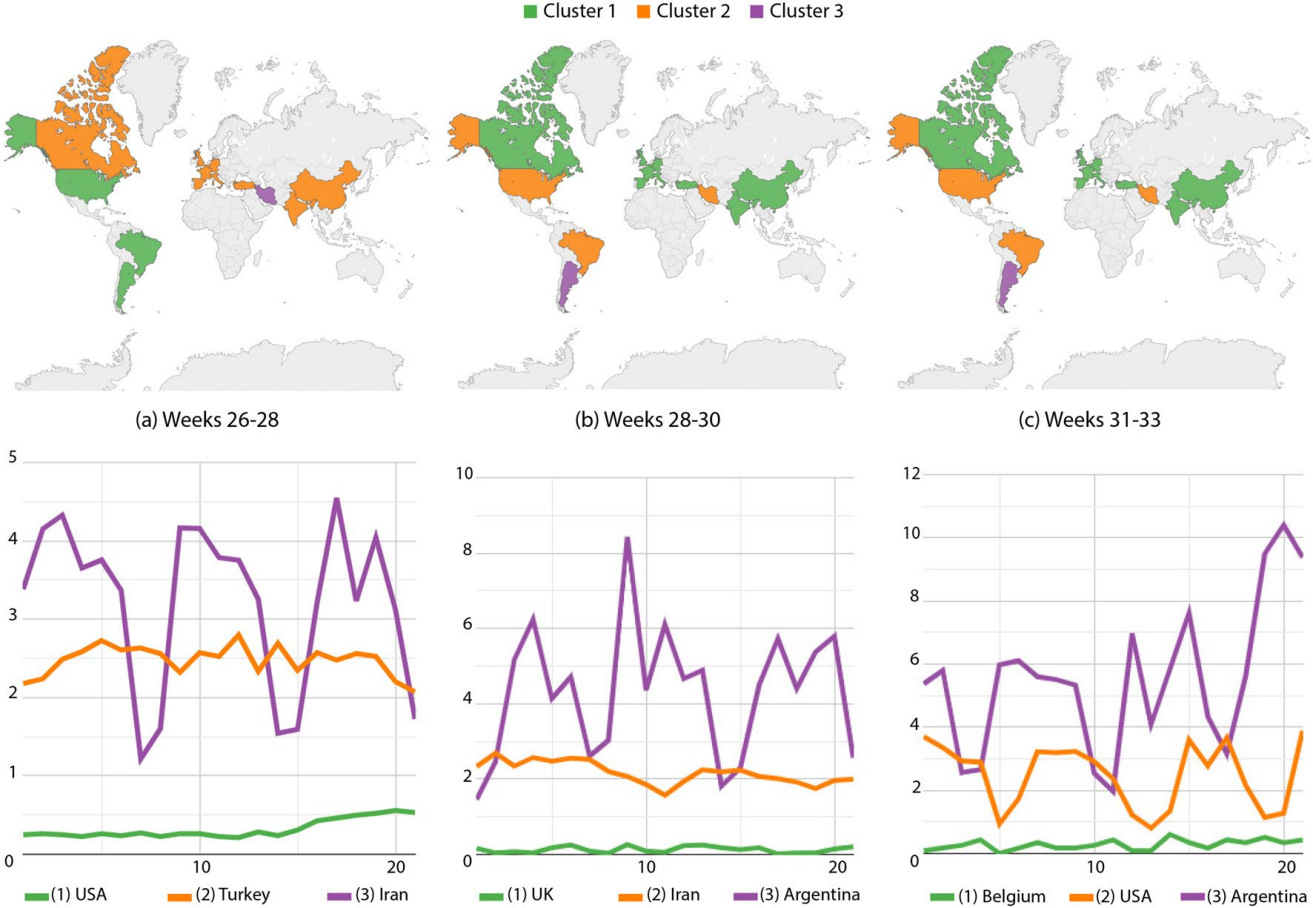
Death cases per million inhabitants: country partitions along weeks 26–33. Top images identify country groups and bottom curves correspond to the infection incidence levels: green, orange and purple correspondingly map the low, medium and high-incidence groups. Curve legends indicate the group descriptor. Top-most maps were generated by using Tableau Desktop-Professional Edition (https://www.tableau.com/, version 20181.20.0213.2110-64 bit), and bottom-most charts were generated by using Google Charts (https://developers.google.com/chart, version 49).

Figure 8a illustrates weeks 26–28, confirming Canada, Turkey, India, China and the Western Europe in the lowest-incidence group; Iran got isolated in the intermediary group, while Brazil, the USA and Argentina were in the worst group. From weeks 28 to 30 (Fig. 8b), Argentina was isolated in the worst group, while Brazil, the USA and Iran were at the intermediary level. Figure 8c shows a similar scenario except due to a greater variance in the intermediary group and some increase of death reports in Argentina.

### Visual transition

**Figure 9:**
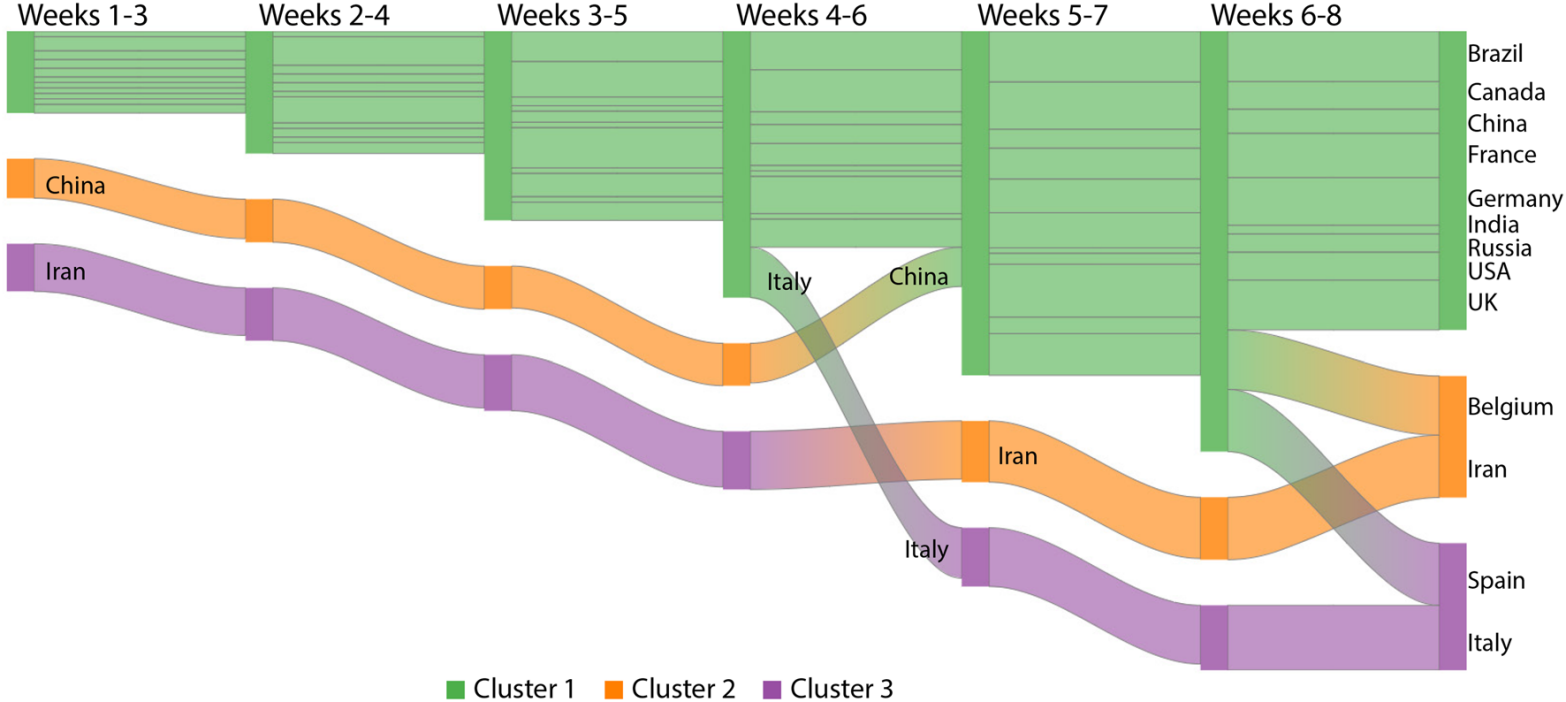
Confirmed cases for the first eight weeks: visualization of the temporal transition of countries between pairs of clusters. Green, orange and purple correspondingly map the low, medium and high-incidence groups. This chart was generated by using Google Charts (https://developers.google.com/chart, version 49).

In Fig. 9, we analyzed how some countries with the greatest numbers of confirmed cases per million inhabitants transitioned along groups. Brazil, Canada, China, France, Germany, India, Russia, USA, UK, Belgium, Iran, Spain and Italy were analyzed. Line widths represent the mean number of confirmed cases within some time window. As new cases are registered, one may notice how countries behave over time.

By assessing Italy, we notice the number of confirmed cases has rapidly increased (line width), leading it from the green (lowest incidence) to the purple cluster (highest incidence) during the first 5 weeks. In weeks 6–8, a similar behavior is noticed with Spain that moves to the same group as Italy. According to Fig. 2c, such countries are characterized by a strong positive trend. Another important information highlighted by the visual transition is the line width of Brazil, France, Germany, the USA, and the UK that got wider as new cases were reported, indicating those countries were moving to high-incidence groups.

**Figure 10:**
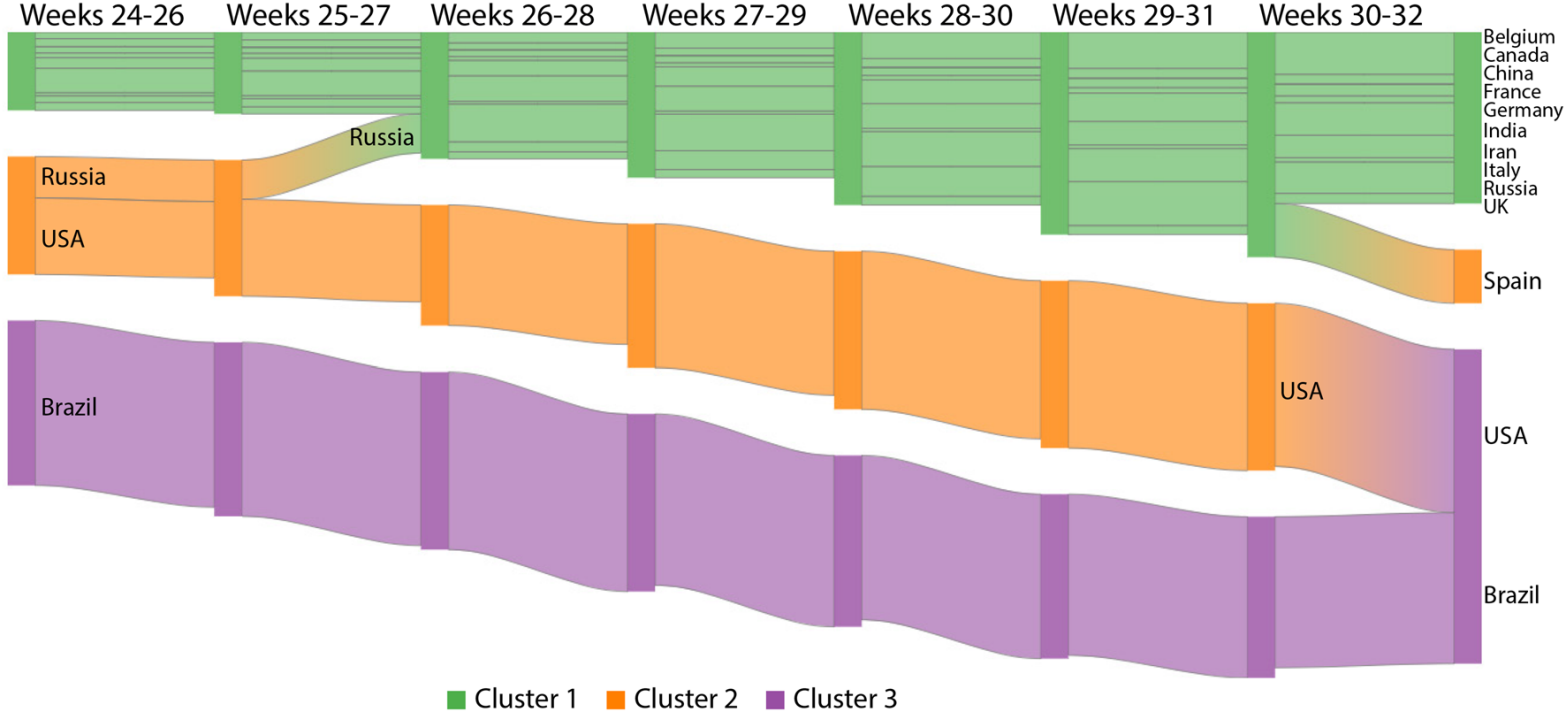
Confirmed cases for weeks 24–32 given no relevant change was observed later: visualization of the temporal transition of countries between pairs of clusters. Green, orange and purple correspondingly map the low, medium and high-incidence groups. This chart was generated by using Google Charts (https://developers.google.com/chart, version 49).

To estimate new waves, we also analyzed some countries by using the visual transition during the last weeks (Fig. 10). One may notice the line width of Spain increases during weeks 30–32, leading it to an intermediary cluster and pointing out the beginning of its second wave. The line widths for Belgium and India have been increasing, calling the attention of their public authorities. Meanwhile, besides Russia moved from the intermediary to the less affected group, its line width still suggests awareness. Another interesting situation is observed for the USA, whose number of confirmed cases increases making it be grouped with Brazil. Indeed, it was an expected behavior due to the agglomeration caused by several protests^21^ and the election run^22,23^.

### Modeling transitions

The trend of confirmed cases is essential to support local public authorities in modeling the probability of a country or region in facing a new wave. From this motivation, we designed a transition index to assess contamination trends detailed in “Country transition index”.

Moreover, our index brings scientific justification to country intervention measurements in an attempt to reduce the disease incidence due to the use of non-pharmacological policies. As a piece of remainder, our index takes a pair of time-consecutive hierarchical clustering partitions to measure the distance ratio of a specific country from its current group to its most probable next one.

**Figure 11:**
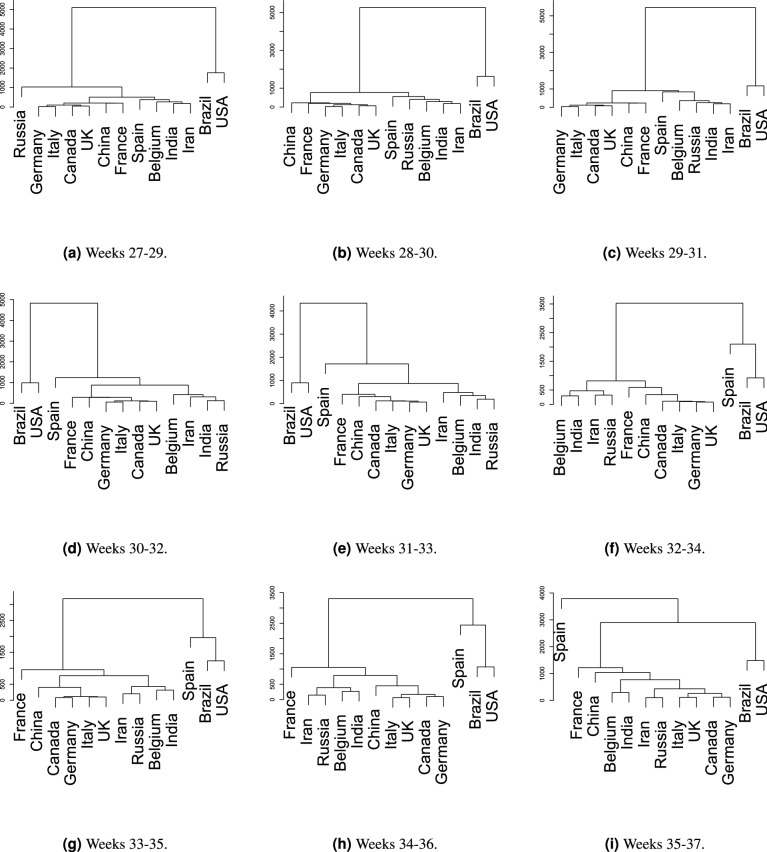
Spain prediction using weeks from 27 to 37 (starting counting weeks just after the first death in Brazil). The software used to cluster the time series and plot the dendrograms was the package *hclust* from R version 3.6.3.

To exemplify how our index captures the temporal transition information as new data is collected, we used last-weeks data to analyze new cases in Spain. In this illustration, the transition index $\mathscr {T}_{X}$ of a country *X* calculates the distance from its current cluster to its closest one (further details about the transition index is provided in “Country transition index”).

In Fig. 11a, Spain is distant $\Delta h = (379 - 265) = 114$ its current cluster composed of Belgium, India, and Iran. Its distance to its closest cluster composed of France, China, the UK, Canada, Italy, and Germany is $\Delta H = (505 - 265) = 240$. From those distances, we calculate the transition index $\mathscr {T}_\text {Spain} = \frac{\Delta h}{\Delta H} = 47.5\%$, assessing the possible move of Spain towards both subgroups. The closer to $100\%$ this index is, the greater is the probability of moving to another group.

We noticed that some transitions between groups happened even without having this index close to $100\%$. This situation is expected once the whole environment is episodic and dynamic^24^, i.e., while analyzing a country, the recorded numbers of others may also change. Such a relation to other countries is illustrated in Fig. 11b, in which the distance from Spain to the subgroup containing Belgium, India, and Iran increases, but Russia gets between them, thus providing a transition index equals to $\mathscr {T}_\text {Spain} = 41.1\%$. It means the number of cases in Spain surpasses Russia’s. Next, Spain gets even far from this subgroup (Fig. 11c), what is corroborated by the transition index $\mathscr {T}_\text {Spain} = 88\%$.

As a consequence, Spain gets closer to the USA and Brazil having the greatest numbers of confirmed cases in such window (Fig. 11d). After calculating the transition indexes from Fig. 11d,e, we get $\mathscr {T}_\text {Spain} = 9\%$ and $\mathscr {T}_\text {Spain} = 23.4\%$, respectively. This strong variation emphasizes the transition that happened in the next dendrogram Fig. 11f, indicating the disease was escalating in Spain thus approaching the numbers of Brazil and the USA.

The next transition indexes of Spain—Fig. 11f–h with $\mathscr {T}_\text {Spain} = 45.2\%$, $\mathscr {T}_\text {Spain} = 37.5\%$, and $\mathscr {T}_\text {Spain} = 61.4\%$, respectively—strongly suggest a new contamination wave and the next COVID-19 epicenter returning to Europe. Finally, as expected, Spain moved away from Brazil and the USA to lead the number of confirmed cases—Fig. 11i. Besides analyzing Spain, we call the readers’ attention back to India, France, and China, whose numbers are strongly increasing.

For example, after calculating the transition index for France, only using the last three time windows (Fig. 11g–i), we obtained $\mathscr {T}_\text {France} = 7.8\%, 10.5\%$, and $21.26\%$, respectively (this last one does not consider China, only the nearby countries as Germany, the UK, Italy, and Belgium), respectively. By keeping China in this last analysis, Fig. 11i, the transition index $\mathscr {T}_\text {France}$ would reduce, once China took a place between France and the group of countries below it. However, the distance between France and its local neighbors was, indeed, increasing, thus suggesting the number of cases was higher as well. Therefore, during the period of our analysis (up to October 7th, 2020), the positive trend supports the conclusion that France is also approaching a new contamination wave.

## Discussion

This research has presented a new approach to analyze the notification evolution of confirmed and death cases per million inhabitants in different countries due to the COVID-19. We have observed that there is a strong motivation to understand and eventually forecast the COVID-19 evolution in a given country by taking into account historical reports from other countries^14–19,25^.

Such observation has called our attention and motivated us to rise a fundamental question: Could we take country “X” to understand the evolution of cases caused by Sars-CoV-2 in another country “Y”? To answer this question, we have designed a new artificial intelligence approach based on unsupervised machine learning methods to perform an exploratory data analysis, without information provided by specialists (e.g. label), to create partitions of countries that minimize intra-cluster and maximize inter-cluster distances.

In summary, the main contributions of our work are the organization of time series, thus better allowing a comparison among different countries, which is a challenge in the COVID-19 scenario, and the transition index. Our approach emphasizes the number of cases of a country is indeed useful to analyze possible outcomes in other regions. When a country is not placed in the same cluster, they cannot be considered somehow similar. In addition to the partition information, we recommend the use of our transition index to calculate eventual country trends over time in an attempt of identifying the next waves and draw public prevention and containment policies. In addition to the contribution to the study of COVID-19, our transition index is also a relevant proposal to future researches in the unsupervised machine learning area due to the possibility of extracting new information from cluster partition.

Finally, our visualization metaphors allow understanding the historical infection path taken by specific countries and estimate new waves using the transition index. In future studies, we plan to include a longer historical series and other demographic and social indicators (i.e., criminality, economy, population density). Additionally, other clustering methods as such as Latent Class Analysis^26,27^ present an attractive alternative to the methods used here.

The main limitations of our analyses are related to the challenges to collect and compare new cases and deaths from different countries. As discussed in the Data Processing section, different monitoring strategies are considered by the affected countries, which may add biases to the analyzed data^28,29^. Furthermore, especially in early phases of the pandemic, the criteria for data collection were not uniform^30,31^, once the virus reaches the countries, and their health systems, at different moments. We emphasize such limitations are not only related to our proposed approach, but also usually faced by data-driven projects in general.

## Methods

### Data processing

The comparison between countries is an essential tool for the control of COVID-19, thus allowing to learn, for example, variations and similarities from different regions, and time trends^28^. However, such a comparison is not an easy task due to the different strategies to collect data, restrain the disease, and report new cases. Therefore, aiming at mitigating these drawbacks, comparisons are only possible by considering that the virus arrives at different moments in every country^29^. Besides that, absolute numbers are incomparable due to different population sizes^29^. Finally, the analysis on cumulative cases might not easily support the identification of local variation, that is, cyclical and seasonal components within short periods of time.

In this sense, we designed our experiments on time series from 186 countries available in COVID-19-CSSE, containing the numbers of confirmed and death cases per million inhabitants. To proceed with our analysis, each of those time series was transformed into daily observations, aiming at supporting the identification of their intrinsic similarities as, for example, local trend and seasonality influences, usually hidden by cumulative analyses. For example, let confirmed cases be organized as $X = \{x_1, x_2, \dots , x_t\}$, in which $x_i$ is the absolute number of cases per million inhabitants registered up to the *i*th day. Then, each series is reorganized to represent the number of cases registered at every individual day, i.e., $\hat{X} = \{\hat{x}_1, \hat{x}_2, \dots , \hat{x}_{t-1}\}$, given $\hat{x}_i = x_{i+1}-x_{i}$ and $1 \le i < t$. Next, all time series are aligned from the first death and confirmed cases to allow the use of historical data to study the disease spreading, once not all countries are homogeneously affected by virus^29^. More details about the importance of the time series alignment is discussed in “Country transition index”.

### Artificial intelligence: unsupervised learning

The recent artificial intelligence researchers have been dedicating a great effort to model the occurrence of new COVID-19 cases. As discussed by Aydin and Yurdakul^32^, such researches are focused on using different algorithm biases to extract useful information and patterns from data in order to examine factors that may affect the number of cases, deaths, and recovered patients. From a carefully search in the literature, we also noticed valuable researches that model COVID-19 data by taking into account the temporal dependencies among their observations^18,19,25,32–35^.

After analyzing such manuscripts, we have realized an important research opportunity that aims at using unsupervised learning to perform an exploratory data analysis on the COVID-19-CSSE dataset looking for similar patterns in different countries over time.

Unsupervised learning looks for data space structures when no label information is available^11^, from which data are organized into partitions (or other structures) to reflect the similarities among objects. Traditional clustering algorithms assume datasets are independent and identically distributed^11^. However, our data has evident dependencies once confirmed cases and deaths result from temporal interactions among people, therefore a different criterion must measure object similarities. In this sense, we employ Dynamic Time Warping (DTW)^36^ to find the best alignment between series and compute their similarities. DTW maps all series elements into one another to reduce their dissimilarities over time. In our experiments, we have considered the Euclidean distance as a pointwise (local) distance function to calculate the warping path^37^.

In this research, we decided to use hierarchical clustering^38–40^ once: their execution is completely deterministic, allowing reproducibility; and they extract patterns under different cluster shapes. Moreover, hierarchical clustering has also been used in other epidemiological applications besides COVID-19, such as chronic inflammatory diseases^41^, airborne infectious diseases^42^, Alzheimer’s Disease^43^, Ebola^44^ and others^45^.

Our algorithm starts with a single cluster per object, then clusters are iteratively merged using a bottom-up approach (agglomerative) until providing a single group containing all objects. At every step, two clusters are merged together using a linkage method^11^. The average-link is used to merge the two nearest clusters based on the mean distance among their inner objects, as defined in Eq. (1), in which $C_p$ and $C_q$ are two clusters, *X* and *Y* are time series belonging to those clusters, function $d(\cdot , \cdot )$ is the DTW method.

1 $$\begin{aligned} dist(C_p, C_q) = \frac{1}{|C_p|\cdot |C_q|}\sum _{\begin{array}{c} \forall X \in C_p \\ \forall Y\in C_q \end{array}} d(X, Y),\; \text {for}\; p \ne q \end{aligned}$$

### Our approach

**Figure 12:**
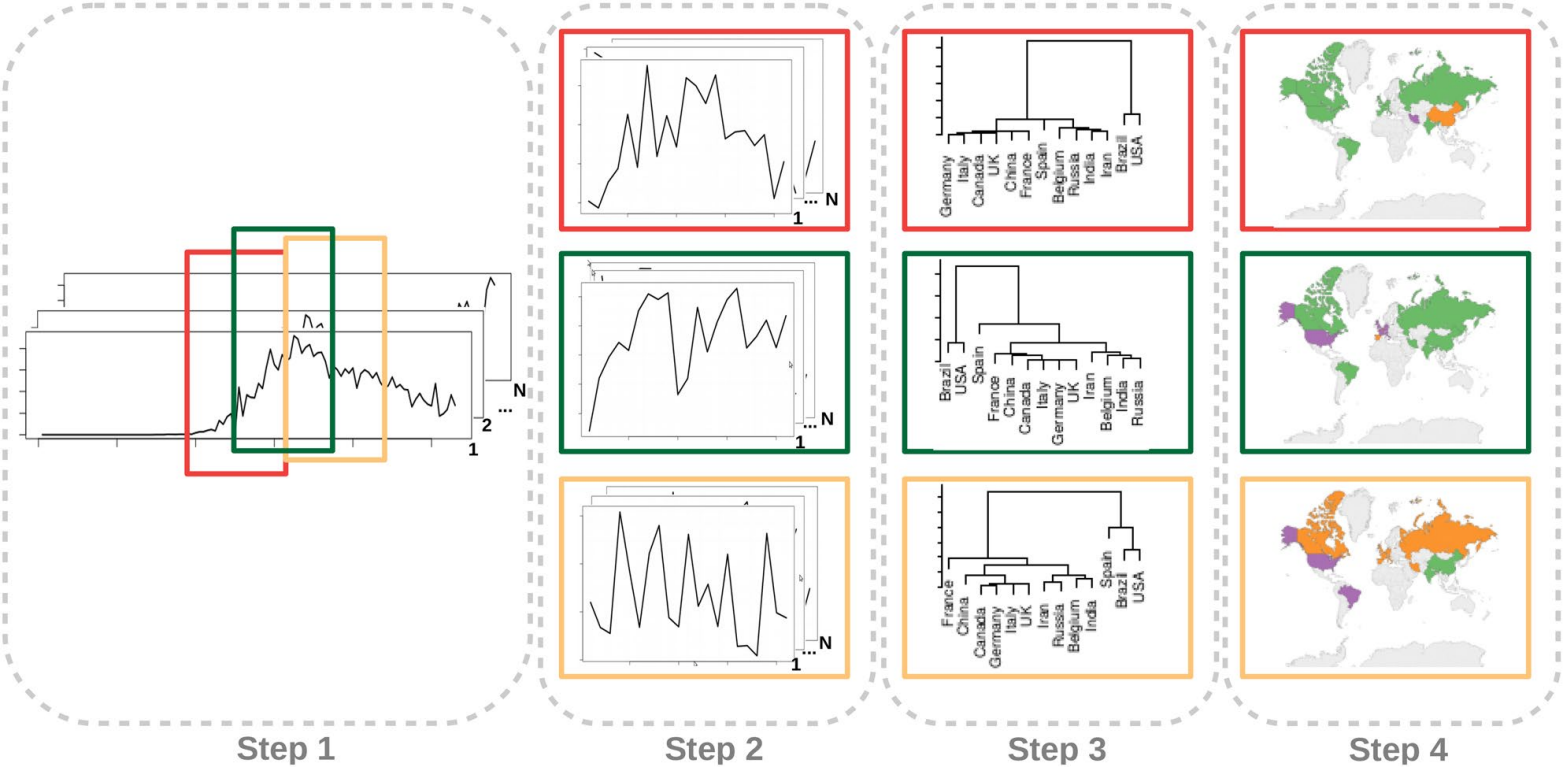
General overview about the proposed approach. Firstly (left-most), the time series are aligned by considering a reference country. Then (middle), we calculated the distance matrix that will be used by the clustering algorithm (right-most).

Our approach is composed of four steps, as illustrated in Fig. 12. Firstly, we analyzed each dataset containing the daily-confirmed cases and deaths per million inhabitants by aligning all time series according to the first record of a given country (left-most plot). The time series alignment is a very important step once all countries are not simultaneously affected by the virus^29^. For example, during the first global wave, the highest number of cases (921) in Italy happened on March 27th, 2020, whereas Brazil was still registering the first deaths. Without aligning their time series, they would never be placed in the same group with higher records, once there is a displacement between the crest points on their waves. Moreover, our goal is not only to analyze the similarities among countries. In turn, our focus is to identify a transition that indicates a given country is moving from a group (e.g. low occurrence of cases) to another (e.g. with a higher number of cases). This is the main reason why we remove from our analysis countries whose the first record happened after some country of interest, that is, this filter gives the idea of analyzing some next disease epicenter depends on past data.

Aiming at illustrating these assumptions, consider the USA as the country of interest. First, we align its first death (recorded on February 29th, 2020) along with the first death in other countries as, for example, in Italy that happened on February 21th, 2020. Then, we can compare whether, after a few days during their first wave, the USA was presenting enough similar behavior to be placed into the same group as Italy, which was the COVID-19 epicenter at that time. In our strategy to compare different countries, by considering this wave-based behavior of the infection, we would not intend to compare whether, after the first death, the USA was going to present a behavior similar to Brazil, whose first death wave started later on March 17th, 2020. The reverse analysis makes sense, though, that is, we can use both the USA and Italy by considering Brazil as a country of interest, thus calculating if Brazil is approaching the USA or Italy. Although we recommend the alignment and filtering processes to compare different countries, the reader can omit them to consider all countries starting from the same day.

Then, we define the time length to support the comparison of evolving behaviors (Step 2), using a sliding window with 3 weeks moving a week forward, forgetting the first 7 observations and including 7 new ones (2-week overlapping). To illustrate this process, consider we are analyzing data from 5 weeks. We start observing the first three windows: weeks 1, 2, and 3. Then, we sliding the window to forget the first week of data and include the next one, thus analyzing weeks 2, 3, and 4. Next, the process is repeated by considering weeks 3, 4, and 5. The windowed analyses, illustrated by the bounding boxes with different colors in the left-most plot of Fig. 12, create the movement captured by our visual analyses and are used by our transition index to perform predictions.

At the third step, DTW is applied on evolving pairs of time series under the same window length, proceeding with the hierarchical clustering and the dendrogram analysis. DTW produces square matrices containing dissimilarity values between all time series pairs. At last, we perform the average-link-based hierarchical clustering on all dissimilarity matrices to build up a dendrogram (Step 3 of Fig. 12). To proceed with the visual metaphor interpretation, we cut the dendrogram at a selected height to form partitions and use such information to color countries (Step 4 of Fig. 12).

### Cluster validity

The quality of clustering partitions is assessed using the mean silhouette $S_\mu$ over all analyzed objects, summarizing the geometric measures of group compactness and separation (more details in^11,46^). The best partition is achieved when $S_\mu$ is maximized, reflecting the minimization of intra and the maximization of inter-cluster distances.

### Country transition index

**Figure 13:**
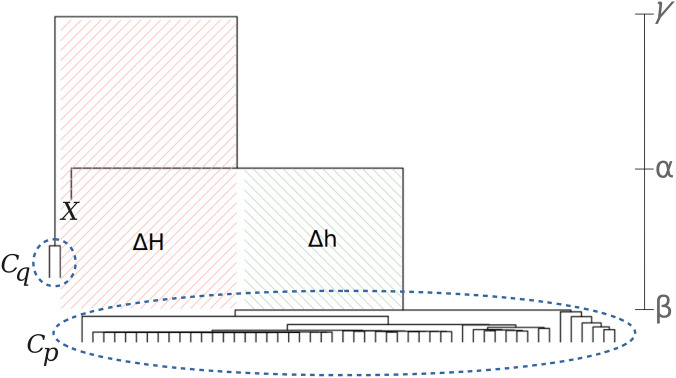
Visual interpretation of $\mathscr {T}_{X}$. In this illustration, the transition index of a country *X* calculates the distance from its current cluster $C_p$ to its closest one $C_q$. For example, if this ratio increases in the next time window, the chance of moving *X* to $C_q$ is greater. The dendrogram clades $\alpha$, $\beta$, and $\gamma$ are used to calculate $\mathscr {T}_{X}$. This figure was generated by using the software Inkscape 1.0.2 available at https://www.inkscape.org.

We designed a country transition index to model historical events and track infection changing points. Let a time series of a specific country be *X* and its two closest groups $C_p$ and $C_q$, $\mathscr {T}_{X}$ measures the transition relation of *X* in form $\mathscr {T}_{X} = \frac{\Delta h}{\Delta H}$ (Fig. 13). In this equation, $\Delta h$ measures the height of the branch that connects the group with *X* (clade $\alpha$) and the lower cluster (clade $\beta$): $|\alpha - \beta |$. In turn, $\Delta h$ calculates the height between the lower (clade $\beta$) and greater (clade $\gamma$) groups by using $|\gamma - \beta |$. Thus, one can identify whether a given country *X* has been moving out $C_p$ towards $C_q$ or the opposite, for $p \ne q$.

This ratio is defined in Eq. (2), in which function *d* is DTW and the groups $C_p$ and $C_q$ were built upon the average-link criterion (Eq. 1). With this, we draw conclusions on country transitions to understand infection trends, being specially useful to highlight new contamination waves.

2 $$\begin{aligned} \mathscr {T}_{X} = \frac{|C_q|\sum _{X' \in C_p}d(X, X')}{|C_p|\left[ \sum _{X' \in C_p}d(X, X') + \sum _{X' \in C_q}d(X, X')\right] } \end{aligned}$$

## Data Availability

An online system with our analyses is available at https://github.com/ricardoarios/hcti. The dataset and source codes used to produce the findings of this study can be found at http://dx.doi.org/10.17632/7tyw5d3ccm.2, an open-source online data repository hosted at Mendeley Data-Rios, Ricardo; Nogueira, Tatiane; Coimbra, Danilo; Lopes, Tiago; Abraham, Ajith; Mello, Rodrigo (2020), “Artificial Intelligence to Model the COVID-19 Country Infection Trends”, Mendeley Data, V2.
